# Platelet-lymphocyte ratio and its dynamic changes predict mortality in septic acute kidney injury patients: a retrospective multi-center study using U.S. database and Chinese hospital data

**DOI:** 10.7717/peerj.20522

**Published:** 2026-01-06

**Authors:** Caihong Liu, Xue Tang, Wei Wei, Yongxiu Huang, Mingjing Guan, Jinglei Ren, Binyu Yang, Ping Fu, Ling Zhang, Yuliang Zhao

**Affiliations:** 1 Department of Nephrology, Institute of Kidney Diseases, West China Hospital of Sichuan University, Chengdu, Sichuan, China; 2 Department of Nephrology, West China Hospital of Sichuan University/West China School of Nursing, Sichuan University, Chengdu, Sichuan, China

**Keywords:** Platelet-lymphocyte ratio, Septic acute kidney injury, Mortality, Mediation analysis

## Abstract

**Background:**

The platelet-to-lymphocyte ratio (PLR), a readily available marker that integrates systemic inflammatory burden and immune competence, has emerged as a cost-effective prognostic biomarker in critical care medicine. Elevated PLR has been linked to adverse outcomes across a spectrum of critical illnesses, yet its utility in predicting prognosis among patients with septic acute kidney injury (AKI) remains undefined. Moreover, little is known about how in-hospital trajectories of PLR influence survival outcomes.

**Method:**

This retrospective study employed data from the Medical Information Mart for Intensive Care IV and West China Hospital of Sichuan University. The primary endpoints were 28-day and 90-day all-cause mortality. The association between baseline PLR/changes in PLR (ΔPLR) and 28-day and 90-day mortality was investigated by survival analysis and Cox proportional hazards models. ΔPLR was calculated as PLR at discharge minus PLR at admission. Patients were stratified based on optimal PLR cut-off values determined from the training cohort, and results were validated internally and externally. Predefined subgroup analyses probed for effect modification across key clinical populations, and mediation analysis was conducted to quantify intermediate variables linking PLR/ΔPLR dynamics to patient outcomes.

**Result:**

A total of 1,478 patients were included in the baseline-PLR cohort and 982 in the ΔPLR cohort. In the training set, an elevated baseline PLR (≥335.44) was associated with significantly higher 28-day mortality (26.56% *vs.* 18.66%, *P* = 0.009) and 90-day mortality (46.48% *vs.* 35.61%, *P* = 0.003). These findings were confirmed in both the internal validation cohort and an external cohort, and remained robust after adjustment for demographic, clinical and laboratory confounders. Conversely, lower ΔPLR also predicted the 28-day (14.81% *vs.* 6.94%, *P* = 0.007; HR = 2.261, *P* = 0.005) and 90-day mortality (24.07% *vs.* 15.41%, *P* = 0.029; HR = 1.702, *P* = 0.017), as well as prolonged hospital stay (28.64 *vs.* 21.64 days, *P* = 0.004). The associations between PLR or ΔPLR and mortality remained robust after adjusting for confounding factors. Subgroup analyses indicated that the prognostic value of PLR was particularly pronounced in non-urinary tract infection patients, and a low baseline PLR conferred a significant survival benefit in male patients. Mediation analysis revealed that changes in white blood cell count (ΔWBC) mediated 27.99% of the association between ΔPLR and 28-day mortality.

**Conclusion:**

Both baseline PLR and its dynamic change during hospitalization may serve as significant predictors of mortality in septic AKI. PLR-based indices could aid in risk stratification and early identification of high-risk patients.

## Introduction

Acute kidney injury (AKI) is a heterogeneous and multifactorial syndrome characterized by sudden deterioration of renal function. It occurred in 10–15% of hospitalized patients, and over 50% of critically ill patients in the intensive care unit (ICU), with septic AKI accounts for 45–70% (Mehta et al., 2016; Hoste et al., 2018; Ostermann et al., 2025). Septic AKI often leads to prolonged ICU and hospital stays, and higher mortality, which ranges from 60–70% (Bellomo et al., 2004; Hofhuis et al., 2013). Though microvascular dysfunction, dysregulated inflammation, and metabolic reprogramming might play roles in the development of septic AKI, the precise mechanisms have not been clearly described; hence, effective and specific interventions for septic AKI are still lacking (De Backer et al., 2011; Wang et al., 2012; Maiden et al., 2016; Gómez, Kellum & Ronco, 2017; Peerapornratana et al., 2019; Liu et al., 2024). Given the notable prevalence, obscure pathophysiology, and poor prognosis, early identification of septic AKI trajectory and timely prevention of poor outcomes are of great importance.

Systemic inflammation plays a crucial role in the progression of septic AKI. Complex interactions among platelets, granulocytes, the vascular endothelium, and invading pathogens finely modulate the inflammatory response through intertwined pro-inflammatory and anti-inflammatory pathways (Li, Huang & Sun, 2025, pp. 2015–2020).

Increasing attention has been directed toward identifying reliable inflammatory markers for septic AKI, particularly those derived from complete blood cell counts due to their accessibility and cost-effectiveness (Liu et al., 2020; Atreya et al., 2023; Yin et al., 2024; Wei et al., 2024; Wei et al., 2025). Among these indices, the neutrophil-to-lymphocyte ratio (NLR) has been widely investigated and recognized as a robust predictor of kidney disease, as it reflects both innate (neutrophil-driven) and adaptive (lymphocyte-mediated) immune responses (Buonacera et al., 2022). Elevated NLR was consistently associated with increased risk and severity of chronic kidney disease (CKD) and erythropoietin resistance in hemodialysis (Carollo et al., 2025b; Carollo et al., 2025a; Li, Huang & Sun, 2025, pp. 2015–2020). Notably, both high NLR and ΔNLR were independently associated with all-cause mortality and disease severity in septic AKI patients (Wei et al., 2023; Wei et al., 2024; Shangguan et al., 2025). NLR outperformed other markers, such as white blood cell (WBC), procalcitonin, and monocyte-to-lymphocyte ratio (MLR) in predicting the prognosis of septic AKI, achieving the highest area under the receiver operating characteristic curve (AUROC) for 30-day mortality prediction (0.618 and 0.624 in two large studies) (Wei et al., 2024; Shangguan et al., 2025). The platelet-to-lymphocyte ratio (PLR) is another clinically available and cost-effective index associated with systemic inflammation. Platelets function not only in hemostatic roles but also as immunomodulators by binding pathogens, releasing cytokines, and forming aggregates with leukocytes (Dewitte et al., 2017; Chen et al., 2020b). Lymphopenia is a hallmark of sepsis-induced immune suppression, whereas platelet activation reflects a pro-thrombotic, pro-inflammatory state; hence PLR implicitly quantifies this immune-thrombotic axis (Iba & Levy, 2018; Gasparyan et al., 2019; Nicolai et al., 2020). PLR has shown prognostic value across a wide range of diseases, including malignancy, cardiovascular disease, and AKI (Sunbul et al., 2014; Li et al., 2015; Zheng et al., 2017; Jeon et al., 2023). Both high NLR (adjusted HR = 1.97) and PLR (adjusted HR = 2.62) were regarded as independent risk factors for all-cause mortality in patients with CKD (Umeres-Francia et al., 2022). However, lower PLR was associated with autologous arteriovenous fistula (AVF) dysfunction (odds ratio 9.97; 95% CI [2.53–39.25]) and positively correlated with AVF patency duration (Rho = 0.254, *p* = 0.002) (Messina et al., 2024). For peritoneal dialysis (PD) patients, multivariate Cox regression analysis showed that the all-cause mortality rate was higher in the high PLR group than in the low PLR group (HR = 1.64) (Liu et al., 2021). Similarly, a study found that the mortality in the high NLR group (18.8 *vs.* 7.5 %) and high PLR group (18.8 *vs.* 7.5 %) increased for hemodialysis individuals. Of note, adjusted Cox regression analysis showed the association of mortality and high NLR was lost, while the significance of the association of high PLR and mortality increased (Yaprak et al., 2016).

In light of the results of these studies, it is reasonable to speculate that the PLR might affect the prognosis of septic AKI. Moreover, to the best of our knowledge, few studies have explored whether dynamic changes in PLR during hospitalization add incremental prognostic information. To address this gap, we conducted a retrospective study from two large cohorts, Medical Information Mart for Intensive Care (MIMIC-IV) and West China Hospital of Sichuan University, to investigate the association between PLR/PLR dynamics with the mortality of septic AKI by internal test and external validation.

## Method

### Study design

We performed a multi-center, retrospective observational study to examine the correlation of the PLR with mortality among septic AKI patients, comprehensively utilizing 1,285 records from the MIMIC-IV v3.0 database and 193 records from patients hospitalized in West China Hospital of Sichuan University between August 2015 and August 2021. This MIMIC-IV database comprises extensive and detailed medical records of over 360,000 patients admitted to an ICU and over 200,000 patients admitted to the emergency department at Beth Israel Deaconess Medical Center, covering the period from 2008 to 2022. The MIMIC database was approved by the institutional review boards of Beth Israel Deaconess Medical Center (2001-P-001699/14), the Massachusetts Institute of Technology (No. 0403000206), and our cohort was approved by West China Hospital of Sichuan University (1.0/2022-1948). The study was conducted in accordance with the Declaration of Helsinki. All patient’s personal information was de-identified for privacy. Owing to the retrospective nature of this study, the patient’s consent was not required. This study was a retrospective observational study, and it is reported based on the Strengthening the Reporting of Observational Studies in Epidemiology (STROBE) guidelines. For model development, MIMIC-IV cases were randomly partitioned (7:3) into a training set and an internal test set. The West China cohort served as an external validation set. The sample sizes for the external validation cohort were determined based on the available patients who met the inclusion criteria during the study periods.

### Population selection

Adult patients diagnosed with septic AKI according to the International Classification of Diseases, Ninth/Tenth Revision diagnosis code (ICD 9/10 code) were considered eligible for inclusion. AKI was defined as an increase in serum creatinine by ≥0.3 mg/dL (≥26.5 μmol/L) within 48 h, or an increase by 50% within 7 days, or urine output <0.5 mL/kg/h for more than 6 h according to the Kidney Disease: Improving Global Outcomes (KDIGO) guideline (Kellum & Lameire, 2013). Sepsis was diagnosed according to the third international consensus definition as an acute change in total Sequential Organ Failure Assessment score ≥ 2 points consequent to the infection. Patients who met the following criteria were excluded: (1) AKI attributable to non-septic etiologies (*e.g.*, obstructive uropathy, nephrotoxin exposure, hypovolaemia without infection); (2) insufficient data for analysis.

### Data collection

Clinical data were extracted as follows. MIMIC-IV records were queried with structured query language (SQL) (PostgreSQL 14.5-1, Navicat Premium 15). The patients’ information of our hospital was extracted from electronic health records (EHR) flowsheet. Variables captured included (1) the demographic information such as gender and age; (2) the comorbidities: hypertension, diabetes mellitus (DM), coronary artery disease (CAD), acute respiratory disease syndrome (ARDS), pneumonia, chronic obstructive pulmonary disease (COPD), tumor, polycystic kidney, multiple organ disease syndrome (MODS), urinary tract infection (UTI), and CKD; (3) baseline laboratory variables from the first and last tests during hospitalization, comprising hemoglobin, leucocyte, lymphocyte, platelet, triglyceride, total bilirubin (TBIL), creatinine, blood urea nitrogen (BUN), uric acid, albumin (ALB), and C-reactive protein (CRP), *etc*. The PLR was calculated as platelet/lymphocyte at admission. The follow-up was achieved from EHRs or by telephone call.

### Clinical outcomes

The principal end-points were all-cause mortality at 28 and 90 days. In analyses that used the baseline PLR, survival time was measured from the date of hospital admission to the date of death or censoring. In models assessing the change in PLR (ΔPLR), survival time accrued from hospital discharge to death or censoring. Secondary end-points comprised (i) hospital length of stay, (ii) ICU length of stay, (iii) incidence of septic shock, (iv) need of invasive mechanical ventilation and (v) initiation of renal replacement therapy (RRT).

### Statistical analysis

Continuous data were expressed as mean ± standard deviation (SD) for normal distributions or median (interquartile range, IQR) for skewed distributions. It was analyzed by *t*-test or Wilcoxon test. For categorical variables, the data of distribution are presented as frequencies (percentages), and compared with chi-square test or Fisher’s exact test, as appropriate. If the proportion of missing values was less than 10%, median substitution was used for data imputation; otherwise, the variable was excluded from analysis.

#### Derivation of PLR cut-off values

Receiver operating characteristic (ROC) curves were constructed to identify optimal thresholds of baseline PLR and ΔPLR for predicting 28-day mortality. The point with the highest Youden index (J = sensitivity + specificity −1) defined the cut-off. ΔPLR was calculated as PLR at discharge minus PLR at admission and therefore analyzed only in patients who survived to discharge. Patients were subsequently stratified into “High PLR”/“Low PLR” groups and “High ΔPLR/Low ΔPLR” groups on the basis of these thresholds.

#### Survival analyses

Survival time was measured from admission to death or last follow-up for baseline PLR, and from discharge to death or last follow-up for ΔPLR. Kaplan–Meier curves with log-rank tests compared survival between groups; patients lost to follow-up were censored at the last known alive date. To minimize interference of crude factors, the prognostic value for the 28-day and 90-day survival status of PLR and ΔPLR were further evaluated using univariate (non-adjusted) and multivariate Cox proportional hazards models, and the results are presented as hazard ratios (HRs) with 95% confidence intervals (CIs). A ratio Cox regression analysis was used while adjusting for covariates in the multivariate models. Model 1 was not adjusted for any covariates. Model 2 were adjusted for baseline covariates that differed significantly (*P* < 0.05) between PLR/ΔPLR strata. Risk factors for poor prognosis of septic AKI identified by previous literature, and clinically relevant confounders based on experience/knowledge were additionally adjusted in multivariate Model 3 (Mansur et al., 2015; Jung et al., 2019; Liu et al., 2020; Jiang et al., 2024; Kang et al., 2024; Sun et al., 2025; Han et al., 2025). To prevent powerlifting, correlated variables were avoided in multivariate models.

#### Secondary outcomes, subgroup analyses, and mediation analysis

Associations between PLR/ΔPLR groups and secondary endpoints (hospital length of stay, ICU length of stay, septic shock, invasive mechanical ventilation and RRT) were examined with multivariable logistic regression, reported as odds ratios (ORs) and 95% CIs. Moreover, subgroup analysis was performed to investigate the association of PLR/ΔPLR and 28-day and 90-day mortality varied across different sub-population, which was clarified by age, gender, hypertension, diabetes, pneumonia, tumor, and CKD. To elucidate inflammatory pathways, the potential mediating role of inflammatory markers were explored in the relation of PLR to mortality in septic AKI patients. Mediation analysis was conducted using the causal mediation framework proposed by Imai, Keele & Tingley (2010) implemented *via* the mediation package in R (version 4.3). PLR was treated as the independent variable, while WBC/ΔWBC, neutrophil/Δneutrophil, and CRP/ΔCRP served as mediators, and 28-day and 90-day mortality as the outcomes. Each mediator was analyzed separately in parallel mediation models. The total effect of PLR on mortality was decomposed into direct and indirect effects, which were quantified as HRs derived from the Cox proportional hazards models. Bias-corrected 95% CIs were obtained using nonparametric bootstrapping with 5,000 resamples (Tingley et al., 2014). All the statistical analyses were performed with R (version 4.3; R Foundation for Statistical Computing, Vienna, Austria). A two-tailed *P* < 0.05 was considered to indicate statistical significance.

## Result

### Baseline characteristics

**Figure 1:**
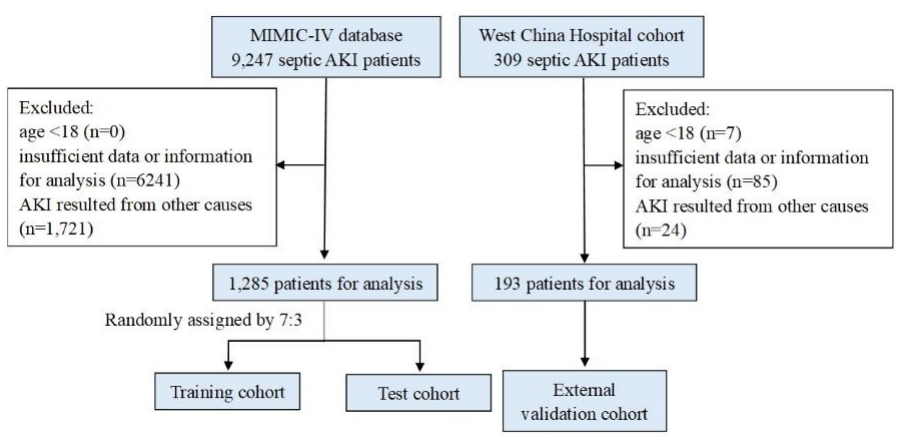
Flow diagram of the study. Abbreviations: MIMIC, Medical Information Mart for Intensive Care; AKI, acute kidney injury.

The flowchart illustrating patient enrollment is presented in Fig. 1. In total, 1,478 patients diagnosed with septic AKI were included in this study, comprising 1,285 individuals from the MIMIC-IV database and 193 from West China Hospital. Of these, 982 patients survived hospitalization (895 from MIMIC-IV and 87 from West China Hospital), making them eligible for analyses involving ΔPLR. Baseline characteristics of patients from the two cohorts are summarized in Table 1. Only hemoglobin had one missing value, and all other variables were complete. The median age was 65 years old, with females accounting for 38.07%. Compared with the West China Hospital cohort, patients from MIMIC database showed higher WBC, neutrophils, platelets, urea nitrogen, creatinine and elevated prevalence of comorbidities of CAD, ARDS, UTI, and CKD. By contrast, lower hemoglobin, albumin, total bilirubin, triglycerides, and likelihood of pneumonia were observed in the MIMIC database. No significant differences were observed in other laboratory parameters or comorbidities between cohorts.

**Table 1:** Baseline characteristics of participants from MIMIC and West China Hospital.

**Variables**	**Total (*n* = 1478)**	**MIMIC (*n* = 1285)**	**West China Hospital (*n* = 193)**	**Statistic**	***P***
**Age, year**	65.00 (53.00, 74.00)	65.00 (54.00, 73.00)	63.00 (48.00, 75.00)	Z = −0.96	0.336
**Gender, n(%)**				*χ*^2^= 2.77	0.096
Female	562 (38.07)	499 (38.83)	63 (32.64)		
Male	916 (61.93)	786 (61.17)	130 (67.36)		
**Serum laboratory test**					
Hemoglobin, g/L	101.00 (85.00, 118.00)	10.00 (8.50, 11.70)	106.00 (84.75, 132.00)	Z = −2.57	0.010
WBC, K/uL	11.11 (7.29, 17.08)	11.60 (7.60, 17.50)	9.12 (6.26, 14.04)	Z = −4.78	<.001
Neutrophils, K/uL	8.95 (5.43, 14.07)	9.23 (5.71, 14.41)	7.38 (4.08, 11.37)	Z = −4.41	<.001
Lymphocytes, K/uL	0.88 (0.52, 1.44)	0.88 (0.51, 1.46)	0.87 (0.56, 1.36)	Z = −0.03	0.979
Platelets, K/uL	185.00 (120.00, 273.50)	192.00 (125.00, 282.00)	153.00 (83.00, 223.00)	Z = −5.27	<.001
Urea nitrogen, mg/dL	28.84 (17.00, 46.00)	30.00 (18.00, 49.00)	17.50 (12.04, 26.52)	Z = −9.58	<.001
Albumin, g/dL	2.80 (2.40, 3.20)	2.80 (2.30, 3.20)	3.13 (2.81, 3.65)	Z = −8.83	<.001
Total bilirubin, umol/L	11.00 (6.84, 22.23)	10.26 (5.13, 22.23)	13.40 (8.50, 22.30)	Z = −3.16	0.002
Triglycerides, mmol/L	0.70 (0.00, 1.81)	0.00 (0.00, 1.81)	1.27 (0.88, 1.82)	Z = −8.60	<.001
Creatinine, mg/dL	1.30 (0.90, 2.00)	1.40 (1.00, 2.10)	0.84 (0.68, 1.06)	Z = −12.80	<.001
**Comorbidities**					
Hypertension, n(%)	462 (31.26)	391 (30.43)	71 (36.98)	*χ*^2^= 3.36	0.067
Received Transplant, n(%)	25 (1.69)	18 (1.40)	7 (3.63)	*χ*^2^= 3.76	0.053
Diabetes Mellitus, n(%)	349 (23.60)	307 (23.89)	41 (21.24)	*χ*^2^= 0.68	0.409
Coronary Artery Disease, n(%)	320 (21.64)	296 (23.04)	24 (12.44)	*χ*^2^= 11.08	<.001
ARDS, n(%)	351 (23.73)	320 (24.90)	30 (15.54)	*χ*^2^= 8.22	0.004
Pneumonia, n(%)	439 (29.68)	291 (22.65)	147 (76.17)	*χ*^2^= 229.79	<.001
COPD, n(%)	159 (10.75)	139 (10.82)	20 (10.36)	*χ*^2^= 0.03	0.852
Tumor, n(%)	246 (16.63)	197 (15.33)	48 (24.87)	*χ*^2^= 10.86	<.001
UTI, n(%)	303 (20.49)	280 (21.79)	23 (11.92)	*χ*^2^= 10.01	0.002
SLE, n(%)	12 (0.81)	9 (0.70)	3 (1.55)	*χ*^2^= 0.65	0.422
CKD, n(%)	189 (12.78)	181 (14.09)	7 (3.63)	*χ*^2^= 16.68	<.001

**Notes.**

Abbreviations MIMIC Medical Information Mart for Intensive Care WBC white blood cell ARDS acute respiratory disease syndrome COPD chronic obstructive pulmonary disease UTI urinary tract infection SLE systemic lupus erythematosus CKD chronic kidney disease

The MIMIC set was then randomly allocated into training and test cohorts with the ratio of 7:3. The comparisons of the two datasets are shown in Table S1. Baseline characteristics between these two subsets were comparable, except for a slightly higher proportion of malignancy in the training cohort. Using ROC analysis in the training set, optimal cut-off values for predicting 28-day mortality were identified as 335.44 for baseline PLR and -46.69 for ΔPLR. The patients were hence divided into the “Low PLR” group (PLR < 335.44, *n* = 643)/“High PLR” group (PLR ≥ 335.44, *n* = 256) and “Low ΔPLR” group (ΔPLR < −46.69, *n* = 108)/“High ΔPLR” group (ΔPLR ≥−46.69, *n* = 519).

### The association of PLR and 28-day and 90-day mortality

**Figure 2:**
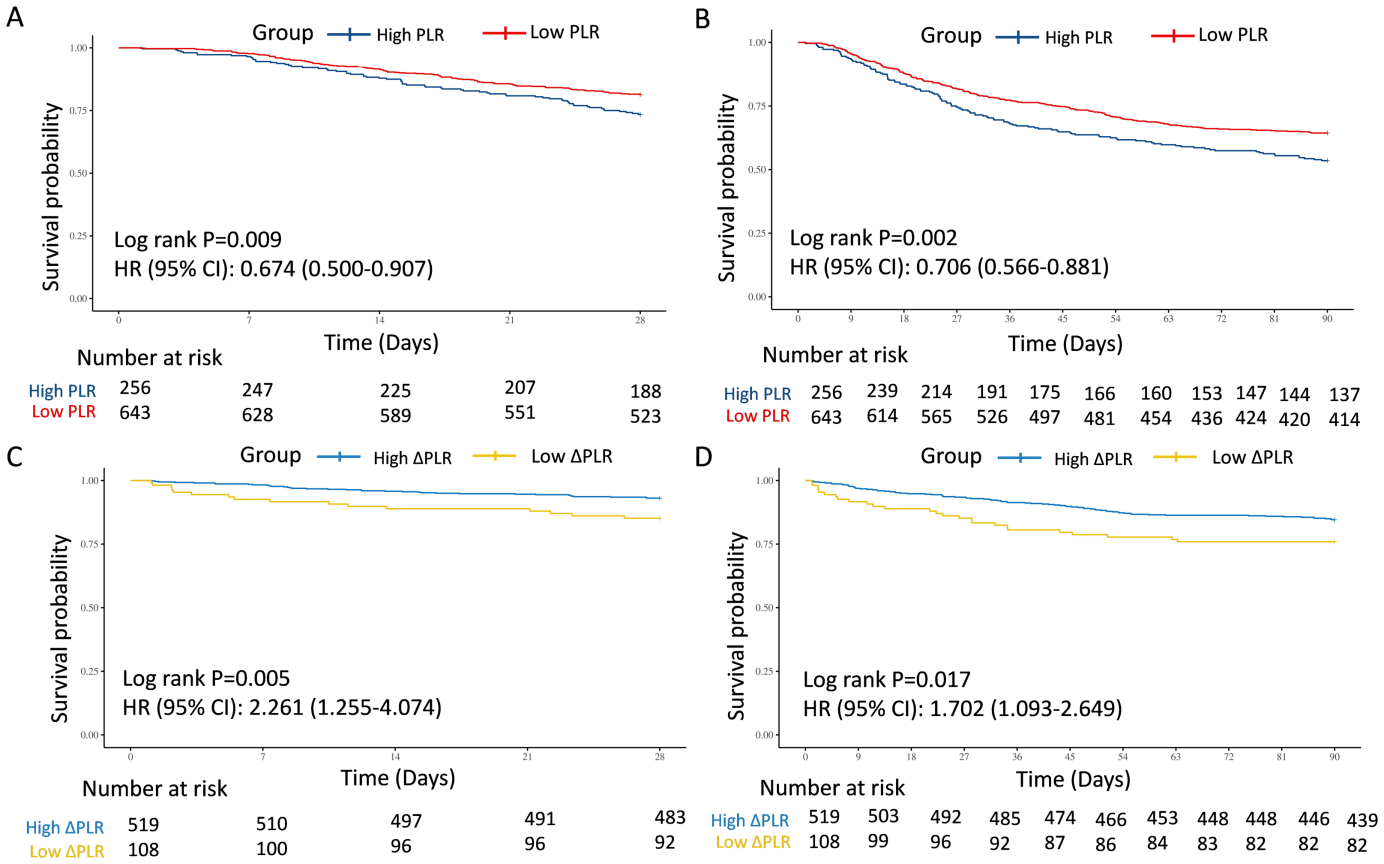
Survival analyses for the association of PLR/ΔPLR and 28-day and 90-day survival in the training cohort. (A) The association of PLR level and 28-day survival. (B) The association of PLR level and 90-day survival. (C) The association of ΔPLR level and 28-day survival. (D) The association of ΔPLR level and 90-day survival. Abbreviations: PLR, platelet-to-lymphocyte ratio; HR, hazard ratio; 95% CI, 95% confidence intervals.

Baseline features comparisons between the two groups were displayed in Table S2. Patients in the High PLR group were older and exhibited higher platelet counts, whereas the Low PLR group had significantly higher WBC counts, lymphocyte counts, and total bilirubin (*P* < 0.05). Kaplan–Meier analyses indicated that patients in the Low PLR group had significantly better survival probabilities at both 28-day (HR = 0.674, 95% CI [0.500–0.907], *P* = 0.009) and 90-day (HR = 0.706, 95% CI [0.566–0.881], *P* = 0.002) follow-ups (Figs. 2A and 2B). Correspondingly, the mortality rates were significantly lower in the Low PLR group compared to the High PLR group at both 28-day (18.66% *vs.* 26.56%, *P* = 0.009) and 90-day (35.61% *vs.* 46.48%, *P* = 0.003) (Table 2). In the univariate model 1, the connection between PLR and 28-day and 90-day mortality was statistically significant (28-day: HR = 0.67, 95% CI [0.50–0.91], *P* = 0.009; 90-day: HR = 0.71, 95% CI [0.57–0.88], *P* = 0.002). In multivariate model 2 adjusted by age, WBC, and TBIL, the PLR remained significantly associated with 28-day and 90-day mortality (28-day: HR = 0.70, 95% CI [0.52–0.94], *P* = 0.019; 90-day: HR = 0.72, 95% CI [0.58–0.90], *P* = 0.004). This predictive value of PLR was also confirmed in model 3 additionally adjusted by gender, hemoglobin, urea nitrogen, concurred with hypertension, diabetes mellitus, CAD, tumor, and CKD (28-day: HR = 0.72, 95% CI [0.53–0.97], *P* = 0.030; 90-day: HR = 0.74, 95% CI [0.59–0.93], *P* = 0.010) (Table 3).

**Figure 3:**
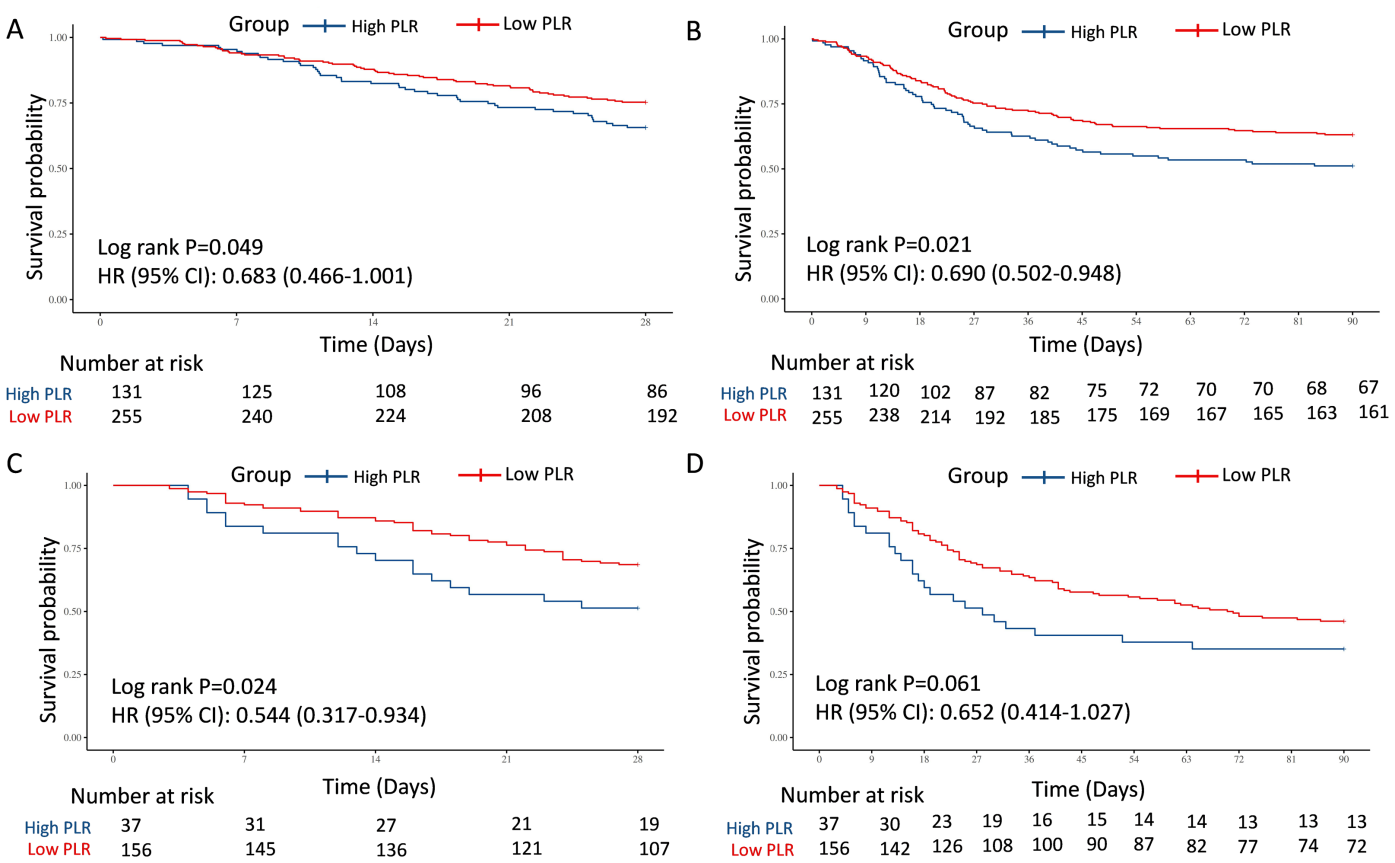
Survival analyses for the association of PLR and 28-day and 90-day survival in the internal test and external validation cohorts. (A) The association of PLR level and 28-day survival in the internal test cohort. (B) The association of PLR level and 90-day survival in the internal test cohort. (C) The association of PLR level and 28-day survival in the external validation cohort. (D) The association of PLR level and 90-day survival in the external validation cohort. Abbreviations: PLR, platelet-to-lymphocyte ratio; HR, hazard ratio; 95% CI, 95% confidence intervals.

In the test and external validation cohort, patients were also clarified into “High PLR” group and “Low PLR” group by the optimal PLR cutoff of 335.44, according to the training cohort. The PLR grade was also a strong predictor for 28-day and 90-day mortality for septic AKI patients, which was verified by 386 individuals in the test cohort from MIMIC (28-day: HR = 0.683, 95% CI [0.466–1.001], *P* = 0.049; 90-day: HR = 0.690, 95% CI [0.502–0.948], *P* = 0.021) (Figs. 3A and 3B) and 193 patients in the external validation cohort from West China Hospital (28-day: HR = 0.544, 95% CI [0.317–0.934], *P* = 0.024; 90-day: HR = 0.652, 95% CI [0.414–1.027], *P* = 0.061) (Figs. 3C and 3D).

To further explore the predictive value, two multivariate models were also performed for test cohort, which were adjusted by crude factors in the training cohort. The result remained robust in Model 2 (28-day: HR = 0.69, 95% CI [0.47–1.00], *P* = 0.058; 90-day: HR = 0.70, 95% CI [0.51–0.97], *P* = 0.032). In Model 3, lower PLR was a potentially protective indicator, but with only borderline statistical significance (28-day: HR = 0.71, 95% CI [0.48–1.06], *P* = 0.097; 90-day: HR = 0.71, 95% CI [0.51–0.99], *P* = 0.047). For external validation with patients from West China Hospital, low PLR also showed potential protective role for survival in Model 2 (28-day: HR = 0.56, 95% CI [0.32–0.98], *P* = 0.042; 90-day: HR = 0.66, 95% CI [0.41–1.05], *P* = 0.081) and Model 3 (28-day: HR = 0.54, 95% CI [0.31–0.93], *P* = 0.025; 90-day: HR = 0.64, 95% CI [0.41–1.02], *P* = 0.060) (Table 3).

### The association of ΔPLR and 28-day and 90-day mortality

With regard to the dynamic changes of PLR, referred to as ΔPLR, patients with Low ΔPLR displayed reduced hemoglobin, TP, ALB, and TBIL, but elevated WBC, Scr, neutrophil counts, platelet, urea nitrogen, and proportion of male (*P* < 0.05) (Table S2). Remarkably, ΔPLR demonstrated strong mortality prediction, with Low ΔPLR associated with 2.3-fold higher 28-day mortality risk and 1.7-fold increased 90-day mortality (28-day: HR = 2.261, 95% CI [1.255–4.074], *P* = 0.005; 90-day: HR = 1.702, 95% CI [1.093–2.649], *P* = 0.017) (Figs. 2C and 2D). The 28-day and 90-day mortality was significantly elevated in the Low ΔPLR group (28-day: 14.81% *vs.* 6.94%, *P* = 0.007; 90-day: 24.07% *vs.* 15.41%, *P* = 0.029) (Table 2). In the univariate model 1, the connection between ΔPLR and 28-day and 90-day mortality was statistically significant (28-day: HR = 2.26, 95% CI [1.25–4.07], *P* = 0.005; 90-day: HR = 1.70, 95% CI [1.09–2.65], *P* = 0.017). In the multivariate model 4 adjusted by gender, hemoglobin, WBC, ALB, TBIL, and Scr, the ΔPLR remained significantly associated with 28-day and 90-day mortality (28-day: HR = 2.47, 95% CI [1.32–4.61], *P* = 0.005; 90-day: HR = 1.61, 95% CI [1.01–2.57], *P* = 0.045). This predictive value of ΔPLR was also confirmed in model 5 additionally adjusted by age, concurred with hypertension, diabetes mellitus, CAD, tumor, and CKD (28-day: HR = 2.57, 95% CI [1.36–4.88], *P* = 0.004; 90-day: HR = 1.66, 95% CI [1.04–2.67], *P* = 0.035) (Table 3).

**Table 2:** The associations of PLR/ΔPLR and clinical outcomes for septic AKI patients.

**Variables**	**PLR**						**ΔPLR**				
	**Total (*n* = 899)**	**High PLR (*n* = 256)**	**Low PLR (*n* = 643)**	**Statistic**	***P***		**Total (*n* = 627)**	**High ΔPLR (*n* = 519)**	**Low ΔPLR (*n* = 108)**	**Statistic**	***P***
**Hospital duration, M (Q**_1_ **, Q**_3_ **)**	22.43 (13.52, 37.02)	23.57 (13.97, 36.17)	21.89 (12.90, 37.41)	Z = −0.57	0.572		22.69 (13.92, 37.85)	21.64 (13.45, 36.20)	28.64 (17.56, 44.64)	Z = −2.88	0.004
**ICU duration, M (Q**_1_ **, Q**_3_ **)**	7.06 (2.57, 15.39)	7.00 (2.14, 15.82)	7.12 (2.80, 15.07)	Z = −0.24	0.811		6.54 (2.42, 15.39)	6.54 (2.35, 15.75)	6.52 (2.57, 13.23)	Z = −0.01	0.996
**28-day death, n(%)**	188 (20.91)	68 (26.56)	120 (18.66)	*χ*^2^= 6.91	0.009		52 (8.29)	36 (6.94)	16 (14.81)	*χ*^2^= 7.30	0.007
**90-day death, n(%)**	348 (38.71)	119 (46.48)	229 (35.61)	*χ*^2^= 9.12	0.003		106 (16.91)	80 (15.41)	26 (24.07)	*χ*^2^= 4.77	0.029
**Invasive mechanical ventilation, n(%)**	320 (35.60)	80 (31.25)	240 (37.33)	*χ*^2^= 2.95	0.086		222 (35.41)	184 (35.45)	38 (35.19)	*χ*^2^= 0.00	0.958
**RRT need, n(%)**	71 (7.90)	18 (7.03)	53 (8.24)	*χ*^2^= 0.37	0.543		45 (7.18)	39 (7.51)	6 (5.56)	*χ*^2^= 0.51	0.473
**Septic Shock, n(%)**	635 (70.63)	189 (73.83)	446 (69.36)	*χ*^2^= 1.76	0.185		394 (62.84)	325 (62.62)	69 (63.89)	*χ*^2^= 0.06	0.804
**Readmitted, n(%)**	594 (66.07)	174 (67.97)	420 (65.32)	*χ*^2^= 0.57	0.449		460 (73.37)	378 (72.83)	82 (75.93)	*χ*^2^= 0.44	0.508

**Notes.**

Abbreviations AKI acute kidney injury PLR platelet-to-lymphocyte ratio ICU intensive care unit RRT renal replacement therapy

**Table 3:** The Cox proportional hazards models of 28-day and 90-day mortality for the training, test, and validation cohorts.

**Cohorts**	**PLR**						**ΔPLR**					
	**Model1**	***P***	**Model2**	***P***	**Model3**	***P***	**Model1**	***P***	**Model4**	***P***	**Model5**	***P***
	**OR (95% CI)**		**OR (95% CI)**		**OR (95% CI)**		**OR (95% CI)**		**OR (95% CI)**		**OR (95% CI)**	
**Training cohort**												
28-day mortality	0.67 (0.50∼0.91)	0.009	0.70 (0.52∼0.94)	0.019	0.72 (0.53∼0.97)	0.030	2.26 (1.25∼4.07)	0.005	2.47 (1.32∼4.61)	0.005	2.57 (1.36∼4.88)	0.004
90-day mortality	0.71 (0.57∼0.88)	0.002	0.72 (0.58∼0.90)	0.004	0.74 (0.59∼0.93)	0.010	1.70 (1.09∼2.65)	0.017	1.61 (1.01∼2.57)	0.045	1.66 (1.04∼2.67)	0.035
**Test cohort**												
28-day mortality	0.68 (0.47∼1.00)	0.049	0.69 (0.47∼1.01)	0.058	0.71 (0.48∼1.06)	0.097	2.69 (1.26∼5.72)	0.007	2.56 (1.18∼5.52)	0.017	2.92 (1.29∼6.60)	0.01
90-day mortality	0.69 (0.50∼0.95)	0.021	0.70 (0.51∼0.97)	0.032	0.71 (0.51∼0.99)	0.047	2.85 (1.48∼5.49)	0.001	2.76 (1.41∼5.39)	0.003	3.06 (1.51∼6.23)	0.002
**External validation cohort**												
28-day mortality	0.54 (0.32∼0.93)	0.024	0.56 (0.32∼0.98)	0.042	0.54 (0.31∼0.93)	0.025	2.59 (0.58∼11.57)	0.196	4.01 (0.75∼21.40)	0.104	4.95 (0.65∼37.53)	0.122
90-day mortality	0.65 (0.41∼1.03)	0.061	0.66 (0.41∼1.05)	0.081	0.64 (0.41∼1.02)	0.060	2.32 (0.71∼7.61)	0.152	2.39 (0.66∼8.66)	0.186	2.20 (0.52∼9.28)	0.281

**Notes.**

Abbreviations PLR platelet-to-lymphocyte ratio OR odd ratio

**Figure 4:**
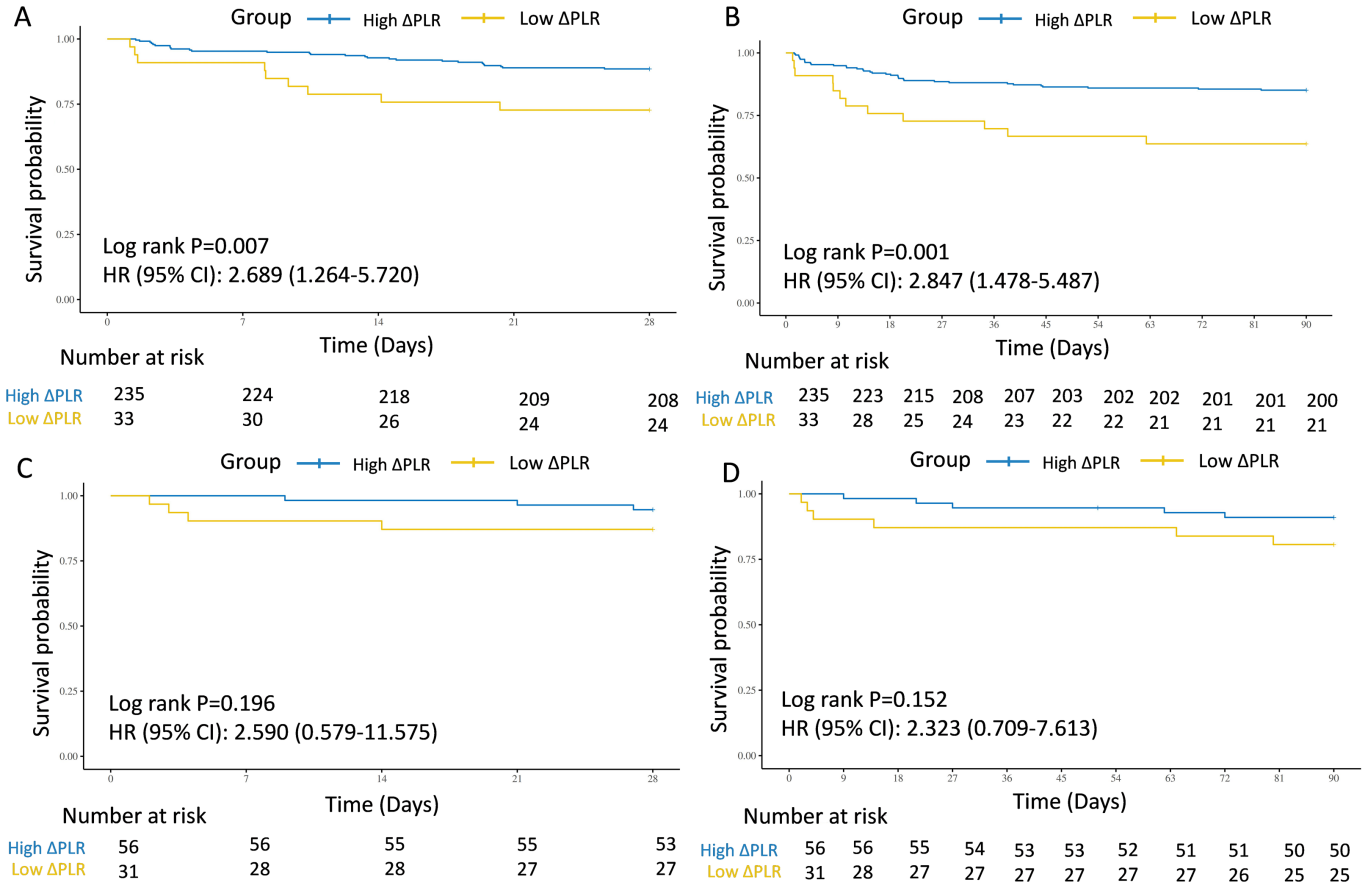
Survival analyses for the association of ΔPLR and 28-day and 90-day survival in the internal test and external validation cohorts. (A) The association of ΔPLR level and 28-day survival in the internal test cohort. (B) The association of ΔPLR level and 90-day survival in the internal test cohort. (C) The association of ΔPLR level and 28-day survival in the external validation cohort. (D) The association of ΔPLR level and 90-day survival in the external validation cohort. Abbreviations: PLR, platelet-to-lymphocyte ratio; HR, hazard ratio; 95% CI, 95% confidence intervals.

In the test and external validation cohort, patients were also clarified into “ΔHigh PLR” group and “ΔLow PLR” group by the optimal PLR cutoff of −46.69, according to the training cohort. The connection between low ΔPLR grade and 28-day and 90-day mortality for septic AKI patients was also significant from 268 individuals in the test cohort from MIMIC (28-day: HR = 2.689, 95% CI [1.264–5.720], *P* = 0.007; 90-day: HR = 2.847, 95% CI [1.478–5.487], *P* = 0.001) (Figs. 4A and 4B), while in the external validation cohort from West China Hospital, despite showing a similar trend, no statistically significant associations were observed (28-day: HR = 2.590, 95% CI [0.579–11.575], *P* = 0.196; 90-day: HR = 2.323, 95% CI [0.709–7.613], *P* = 0.152) (Figs. 4C and 4D).

Furthermore, two multivariate models were also performed for the test cohort, which were adjusted by crude factors in the training cohort. The results were similar in Model 4 (28-day: HR = 2.56, 95% CI [1.18–5.52], *P* = 0.017; 90-day: HR = 2.76, 95% CI [1.41–5.39], *P* = 0.003) and Model 5 (28-day: HR = 2.92, 95% CI [1.29–6.60], *P* = 0.010; 90-day: HR = 3.06, 95% CI [1.51–6.23], *P* = 0.002). For external validation with patients from West China Hospital, given the limited sample size, low ΔPLR also did not showed significant predictive role for mortality in Model 4 (28-day: HR = 4.01, 95% CI [0.75–21.40], *P* = 0.104; 90-day: HR = 2.39, 95% CI [0.66–8.66], *P* = 0.186) and Model 5 (28-day: HR = 4.95, 95% CI [0.65–37.53], *P* = 0.122; 90-day: HR = 2.20, 95% CI [0.52–9.28], *P* = 0.281) (Table 3).

### Prognostic effects of PLR/ΔPLR based on subgroup analyses

We performed subgroup analyses of the association between PLR at admission and primary outcomes in septic AKI patients. The prognostic utility of the PLR for predicting mortality was meticulously evaluated across various patient subgroups, including age, gender, presence of hypertension, DM, CAD, pneumonia, tumor, UTI, and CKD. Subgroup stratification revealed enhanced PLR prognostic utility in non-UTI patients for 90-day mortality. Male was especially protected by low PLR for both 28-day and 90-day death (Table 4).

**Table 4:** Subgroup analysis for the predictive value of PLR/ΔPLR on 28-day and 90-day mortality.

**Variables**	**PLR**			**28-day**			**90-day**			**ΔPLR**			**28-day**			**90-day**		
	**n (%)**	**High PLR**	**Low PLR**	**HR (95% CI)**	***P***	**P for interaction**	**HR (95% CI)**	***P***	**P for interaction**	**n (%)**	**High ΔPLR**	**Low ΔPLR**	**HR (95% CI)**	***P***	**P for interaction**	**HR (95% CI)**	***P***	**P for interaction**
**All patients**	899 (100.00)	68/256	120/643	0.67 (0.50∼0.91)	0.009		0.71 (0.57∼0.88)	0.002		627 (100.00)	36/519	16/108	2.26 (1.25∼4.07)	0.005		1.70 (1.09∼2.65)	0.018	
**Gender**						0.031			0.040						0.341			0.220
Female	347 (38.60)	23/106	53/241	1.02 (0.62∼1.66)	0.940		0.94 (0.66∼1.33)	0.722		230 (36.68)	17/181	7/49	1.61 (0.67∼3.88)	0.288		1.21 (0.60∼2.47)	0.591	
Male	552 (61.40)	45/150	67/402	0.52 (0.35∼0.75)	<.001		0.58 (0.44∼0.78)	<.001		397 (63.32)	19/338	9/59	2.87 (1.30∼6.35)	0.009		2.15 (1.22∼3.79)	0.008	
**Age**						0.803			0.842						0.815			0.815
<65	450 (50.06)	21/108	52/342	0.75 (0.45∼1.24)	0.264		0.72 (0.50∼1.02)	0.065		332 (52.95)	14/280	5/52	1.99 (0.72∼5.53)	0.186		1.55 (0.74∼3.24)	0.242	
≥65	449 (49.94)	47/148	68/301	0.69 (0.48∼1.00)	0.052		0.75 (0.57∼1.00)	0.05		295 (47.05)	22/239	11/56	2.30 (1.11∼4.74)	0.024		1.72 (0.99∼3.00)	0.054	
**Hypertension**						0.671			0.461						0.530			0.256
No	619 (68.85)	51/180	84/439	0.65 (0.46∼0.92)	0.015		0.67 (0.52∼0.87)	0.002		414 (66.03)	24/345	9/69	1.94 (0.90∼4.17)	0.09		1.41 (0.80∼2.49)	0.235	
Yes	280 (31.15)	17/76	36/204	0.75 (0.42∼1.34)	0.331		0.81 (0.53∼1.26)	0.353		213 (33.97)	12/174	7/39	2.84 (1.12∼7.21)	0.028		2.38 (1.16∼4.88)	0.018	
**DM**						0.552			0.843						0.336			0.913
No	690 (76.75)	53/195	91/495	0.64 (0.46∼0.90)	0.010		0.72 (0.55∼0.92)	0.01		484 (77.19)	27/400	14/84	2.62 (1.37∼5.00)	0.003		1.68 (1.01∼2.78)	0.044	
Yes	209 (23.25)	15/61	29/148	0.80 (0.43∼1.50)	0.493		0.69 (0.44∼1.08)	0.1		143 (22.81)	9/119	2/24	1.15 (0.25∼5.32)	0.859		1.79 (0.71∼4.50)	0.218	
**CAD**						0.835			0.646						0.863			0.101
No	690 (76.75)	49/189	92/501	0.69 (0.49∼0.97)	0.035		0.73 (0.56∼0.94)	0.016		490 (78.15)	31/408	13/82	2.24 (1.17∼4.28)	0.015		2.09 (1.28∼3.42)	0.003	
Yes	209 (23.25)	19/67	28/142	0.65 (0.36∼1.16)	0.144		0.65 (0.42∼1.01)	0.057		137 (21.85)	5/111	3/26	2.56 (0.61∼10.70)	0.199		0.79 (0.27∼2.29)	0.660	
**Pneumonia**						0.438			0.796						0.856			0.828
No	691 (76.86)	49/196	92/495	0.72 (0.51∼1.02)	0.065		0.72 (0.55∼0.93)	0.013		501 (79.90)	27/417	12/84	2.32 (1.17∼4.57)	0.015		1.66 (1.01∼2.74)	0.047	
Yes	208 (23.14)	19/60	28/148	0.55 (0.31∼0.98)	0.044		0.66 (0.44∼1.01)	0.057		126 (20.10)	9/102	4/24	2.04 (0.63∼6.64)	0.234		1.86 (0.72∼4.79)	0.199	
**Tumor**						0.350			0.411						0.581		0.846
No	773 (85.98)	57/217	97/556	0.64 (0.46∼0.88)	0.007		0.68 (0.54∼0.87)	0.002		547 (87.24)	27/457	12/90	2.38 (1.21∼4.70)	0.012		1.61 (0.96∼2.68)	0.069	
Yes	126 (14.02)	11/39	23/87	0.92 (0.45∼1.89)	0.826		0.87 (0.51∼1.47)	0.598		80 (12.76)	9/62	4/18	1.62 (0.50∼5.27)	0.421		1.77 (0.72∼4.35)	0.211	
**UTI**						0.080			0.012						0.583			0.123
No	705 (78.42)	62/203	98/502	0.60 (0.44∼0.83)	0.002		0.61 (0.48∼0.78)	<.001		487 (77.67)	28/406	13/81	2.47 (1.28∼4.77)	0.007		2.08 (1.26∼3.44)	0.004	
Yes	194 (21.58)	6/53	22/141	1.41 (0.57∼3.48)	0.454		1.31 (0.76∼2.25)	0.332		140 (22.33)	8/113	3/27	1.64 (0.43∼6.17)	0.467		0.89 (0.34∼2.32)	0.805	
**CKD**						0.283			0.318						0.995			0.103
No	779 (86.65)	55/213	109/566	0.72 (0.52∼0.99)	0.044		0.74 (0.58∼0.94)	0.014		544 (86.76)	28/453	16/91	3.08 (1.67∼5.70)	<.001		2.01 (1.26∼3.20)	0.003	
Yes	120 (13.35)	13/43	11/77	0.44 (0.20∼0.98)	0.045		0.53 (0.30∼0.95)	0.032		83 (13.24)	8/66	0/17	0.00 (0.00∼Inf)	0.999		0.55 (0.12∼2.44)	0.431	

**Notes.**

Abbreviations PLR platelet-to-lymphocyte ratio DM diabetes mellitus CAD coronary artery disease UTI urinary tract infection CKD chronic kidney disease

### Mediation analysis of PLR/ΔPLR on 28-day and 90-day mortality

**Figure 5:**
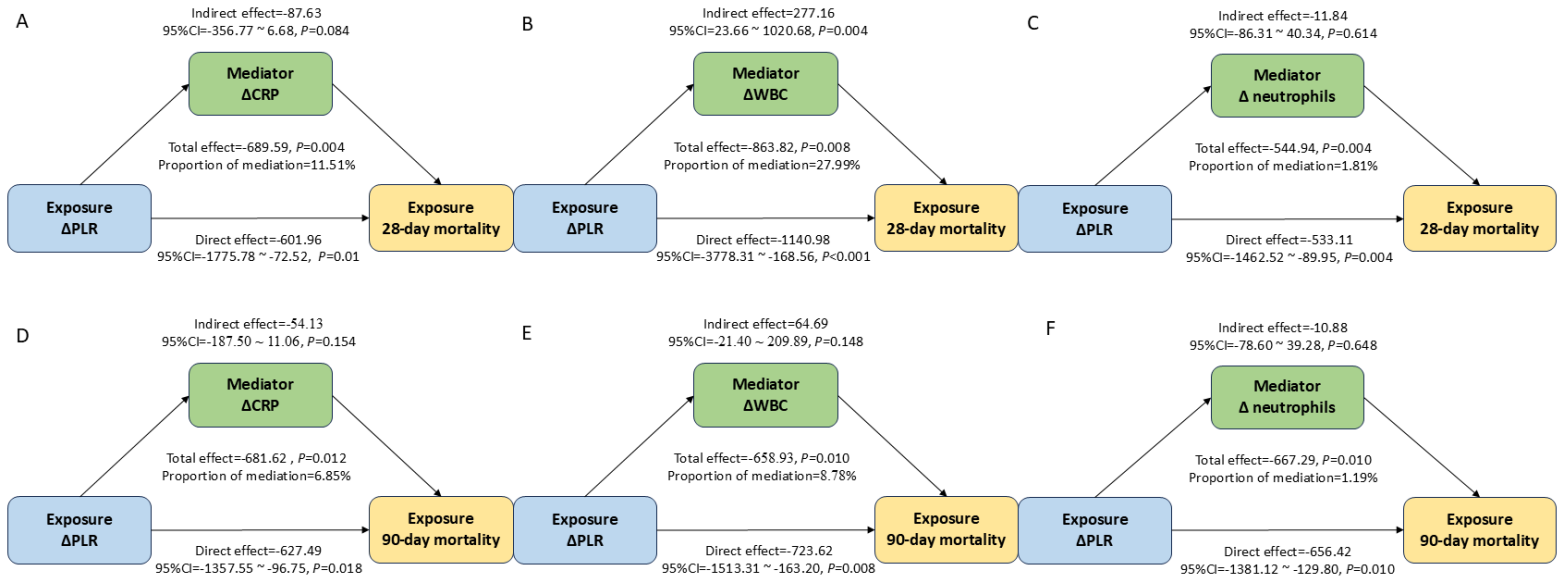
Analysis of the mediation by ΔCRP (A), ΔWBC (B), and Δ neutrophils (C) of the association of ΔPLR and 28-day mortality; by ΔCRP (D), ΔWBC (E), and Δ neutrophils (F) of the association of ΔPLR and 90-day mortality. Abbreviations: PLR, platelet-to-lymphocyte ratio; HR, hazard ratio; 95% CI, 95% confidence intervals; CRP, C-reactive protein; WBC, white blood cell.

This study further assessed the regulating role of inflammation-associated biomarkers:

CRP/ΔCRP, WBC/ΔWBC, and neutrophils/Δneutrophils in the association between PLR/ /ΔPLR and 28-day mortality in the septic AKI patients. This study found that ΔWBC mediated 27.99% of the association between ΔPLR and 28-day mortality (Fig. 5). However, these inflammation factors didn’t serve as the mediator for the impact of ΔPLR on 90-day mortality (Fig. 5). The regulating role of CRP, WBC, and neutrophils was not observed between PLR level and both 28-day and 90-day mortality (Fig. S1).

### Association between PLR/ΔPLR and secondary outcomes

Apart from mortality, low ΔPLR correlated with prolonged hospitalization duration (28.64 *vs.* 21.64 days, *P* = 0.004), but without significant connection with the length of ICU stay (Table 2). But neither univariate nor multivariate logistics analyses demonstrated substantial associations between PLR/ΔPLR and septic shock, RRT need, invasive mechanical ventilation, or readmission proportions (all *P* > 0.05) (Tables 2, 5.

**Table 5:** The multivariate logistic regression analysis of PLR/ΔPLR on clinical outcomes.

**Variables**	**PLR**						**ΔPLR**					
	**Model1**	***P***	**Model2**	***P***	**Model3**	***P***	**Model1**	***P***	**Model4**	***P***	**Model5**	***P***
	**OR (95% CI)**		**OR (95% CI)**		**OR (95% CI)**		**OR (95% CI)**		**OR (95% CI)**		**OR (95% CI)**
**Septic shock**	0.80 (0.58∼1.11)	0.185	0.76 (0.55∼1.06)	0.106	0.77 (0.55∼1.08)	0.129	1.06 (0.69∼1.63)	0.804	0.82 (0.51∼1.30)	0.396	0.81 (0.51∼1.29)	0.380
**RRT need**	1.19 (0.68∼2.07)	0.544	1.06 (0.60∼1.88)	0.830	1.13 (0.63∼2.02)	0.692	0.72 (0.30∼1.76)	0.475	0.39 (0.13∼1.18)	0.095	0.37 (0.12∼1.15)	0.087
**ICU transfer**	1.13 (0.84∼1.52)	0.430	1.08 (0.80∼1.45)	0.627	1.09 (0.80∼1.47)	0.585	1.10 (0.70∼1.73)	0.688	1.04 (0.65∼1.68)	0.858	1.06 (0.66∼1.70)	0.820
**Invasive mechanical ventilation**	1.31 (0.96∼1.78)	0.086	1.26 (0.92∼1.72)	0.149	1.27 (0.92∼1.74)	0.145	0.99 (0.64∼1.53)	0.958	1.06 (0.67∼1.67)	0.816	1.08 (0.68∼1.72)	0.748
**Readmission**	0.89 (0.65∼1.21)	0.449	0.92 (0.67∼1.26)	0.610	0.96 (0.69∼1.32)	0.788	1.18 (0.73∼1.90)	0.508	1.20 (0.73∼2.00)	0.473	1.23 (0.73∼2.06)	0.437

**Notes.**

Abbreviations PLR platelet-to-lymphocyte ratio RRT renal replacement therapy ICU intensive care unit

## Discussion

In this study, we found that a higher baseline PLR was a strong predictor of 28-day and 90-day mortality in patients with septic AKI, as demonstrated in both the internal and external validation cohorts. Subgroup analysis revealed that the prognostic value of PLR was more pronounced in non-UTI patients for 90-day mortality. Interestingly, male patients appeared to benefit more from a lower baseline PLR. In contrast, a lower ΔPLR during hospitalization was associated with poorer prognosis, although this finding was not confirmed in the external validation cohort. The external validation result should be interpreted with caution, as it was inconclusive and likely underpowered, possibly due to the limited sample size and population heterogeneity between the US and Chinese cohorts. Future multicenter, prospective studies are warranted to confirm this association. The potential prognostic role of ΔPLR may be partially mediated by ΔWBC. Furthermore, a lower ΔPLR was significantly associated with prolonged length of hospital stay.

The PLR, proposed as a surrogate marker of systemic inflammation, has emerged as a prognostic indicator across a broad spectrum of diseases. In oncology, a large cohort study involving 8,759 cancer patients demonstrated a positive correlation between elevated PLR and increased mortality (Proctor et al., 2011). Similarly, in cardiovascular disease, PLR was identified as an independent predictor of long-term mortality following non-ST segment elevation myocardial infarction (Azab et al., 2012). In the context of sepsis, a retrospective cohort study using the MIMIC database, which included 5,537 patients, found that higher PLR levels were significantly associated with increased mortality (OR 1.29; 95% CI [1.09–1.53]) (Shen, Huang & Zhang, 2019). Beyond sepsis, PLR has also shown prognostic relevance in kidney-related conditions. Specifically, in both hemodialysis and peritoneal dialysis populations, patients with higher PLR exhibited greater all-cause mortality compared to those with lower PLR (Yaprak et al., 2016). In patients undergoing primary percutaneous coronary intervention, Velibey et al. (2017) reported that elevated PLR independently predicted the risk of contrast-induced AKI. Moreover, PLR also predicted AKI following cardiac surgery with cardiopulmonary bypass (He & Zhou, 2021). Focusing specifically on septic AKI, Chen et al. demonstrated a significant correlation between PLR and 28-day mortality using Spearman correlation analysis (Chen et al., 2020a). Collectively, these findings underscore the prognostic potential of PLR in both inflammatory and renal pathologies. Given its accessibility and clinical relevance, we stratified patients by PLR levels in our study and further explored the association between PLR and mortality in septic AKI patients. However, most of the aforementioned studies were limited by single-center designs, relatively small sample sizes, and a predominant focus on baseline PLR values, without consideration of its dynamic changes over the course of illness.

To address these limitations and enhance the generalizability of findings, we leveraged data from two independent cohorts, including both internal and external validation sets. The study found that baseline PLR strongly predicted 28-day and 90-day mortality in the training cohort from the MIMIC database, which was also been confirmed by internal test and external validation from West China Hospital of Sichuan University. This multicohort approach allowed us to confirm the prognostic value of baseline PLR in a broader septic AKI population. Although it is increasingly recognized, the underlying biological mechanisms remain to be fully elucidated. One proposed explanation involves the stage-dependent immunological shift that occurs during sepsis, in which the dynamic balance between pro-inflammatory and anti-inflammatory responses is critical for both disease progression and organ recovery (Liu et al., 2024). In the acute phase of sepsis, platelets are actively involved in immune responses by releasing inflammatory cytokines and directly interacting with pathogens and immune cells. These actions amplify the systemic inflammatory response, contributing to endothelial injury, microcirculatory dysfunction, and ultimately organ damage (Azab et al., 2012; Cho et al., 2014; Kim et al., 2015; Nording, Seizer & Langer, 2015). Conversely, a reduction in circulating lymphocytes, a hallmark of sepsis-induced immunosuppression, is associated with impaired host defense and a higher risk of secondary infections. Therefore, an elevated PLR may reflect a state of immune dysregulation, characterized by excessive inflammation (platelet activation) and inadequate immune surveillance (lymphopenia). This imbalance may hinder the timely transition from the hyperinflammatory phase to a reparative, anti-inflammatory phase, thereby delaying renal recovery and worsening prognosis (Chen et al., 2020a). In line with this mechanistic hypothesis, our study demonstrated that higher baseline PLR was significantly associated with increased 28-day and 90-day mortality, although it was not significantly linked to other clinical outcomes such as ICU length of stay. These findings suggest that PLR may serve as a marker of immune imbalance rather than a general severity index, and may be particularly useful in identifying patients at risk for poor long-term outcomes.

More importantly, we introduced and analyzed the ΔPLR during hospitalization—an aspect not previously explored in this clinical context. By stratifying patients among those who survived hospitalization according to ΔPLR levels, we aimed to capture the prognostic significance of PLR dynamics, which may reflect underlying immune recovery or persistent dysregulation, thereby providing novel insights into disease progression and long-term outcomes. In this study, we observed that patients with lower ΔPLR had a significantly worse prognosis compared to those with higher ΔPLR, suggesting that the trajectory of PLR during hospitalization may reflect distinct immune recovery profiles in septic AKI. The low ΔPLR (ΔPLR < −46.69) means the PLR decreases relatively largely compared to the baseline level, suggesting continued thrombocytopenia or excessive, dysregulated lymphocyte expansion (Venet & Monneret, 2018). The non-functional immune reconstitution might lead to failure renal recovery or host defense (Delano & Ward, 2016). On the contrary, a relatively stable or rising PLR (ΔPLR > −46.69) may represent an appropriate resolution phase in which platelet counts normalize and lymphocyte recovery is controlled. The rebalance of platelets and lymphocytes signifies a shift toward immune homeostasis (Tang, Huang & McLean, 2010; Van der Poll et al., 2017). This immune trajectory likely contributes to the repair of damaged organs and a better prognosis. Therefore, ΔPLR may serve not only as a dynamic biomarker of inflammation but also as an indirect indicator of immune system reconstitution during septic AKI, providing valuable insight into patient outcomes beyond static measurements. Notably, we also found that ΔWBC mediated 27.99% of the association between ΔPLR and 28-day mortality. This mediating role of ΔWBC reinforces the notion that ΔPLR captures not only platelet and lymphocyte dynamics but also broader immune-inflammatory interactions.

The limitations of this study should be acknowledged. First, despite the inclusion of both internal and external cohorts, the data were derived from retrospective observational databases, which may introduce residual confounding and selection bias. Notably, the cohorts from two centers differed in some baseline characteristics. The MIMIC-IV cohort exhibited higher levels of inflammatory and renal function markers and a higher prevalence of comorbidities, suggesting a population with greater illness severity and metabolic burden. The Chinese cohort had higher hemoglobin and albumin levels, and a greater incidence of pneumonia reflecting potential variations in disease phenotype and nutritional status. The heterogeneity in population demographics, clinical practice, and disease profiles across regions may limit the generalizability of our findings, the consistent direction of associations between PLR and mortality across both cohorts supports the robustness of our results. Future multicenter prospective studies involving more diverse populations are warranted to further validate and extend these observations. Second, although PLR and ΔPLR were associated with mortality, their mechanistic links to immune and inflammatory pathways were inferred rather than directly measured, and lack validation by functional immunologic markers. Third, certain clinically relevant variables, such as specific infection sources, pathogen types, and immunosuppressive medication use, were not uniformly available, which may affect the generalizability of the findings. Moreover, body-mass index (BMI) and total protein were excluded for analysis for a missing proportion of more than 10%. This exclusion might have led to residual confounding, as both nutritional and metabolic status could influence the prognosis of septic AKI. Although missing data were minimal in the selected cohort, median substitution was applied for hemoglobin. This simple imputation method may also introduce minor bias. Lastly, while ΔPLR showed potential prognostic value, it should be considered with caution, since the prognostic value of ΔPLR has not been validated by external data, which was possibly due to the limited sample size in the external validation cohort. Future large-scale cohorts with more complete datasets are required to confirm the findings.

## Conclusion

In conclusion, this study demonstrated that baseline PLR was significantly associated with mortality in patients with septic AKI across both cohorts. By contrast, while ΔPLR showed a potential association with outcomes, this finding was not statistically confirmed in the external validation. Furthermore, ΔWBC partially mediated the association between ΔPLR and mortality, suggesting an interplay between immune recovery and systemic inflammation. These findings underscore the prognostic potential of PLR as a simple, accessible, and integrative biomarker for risk stratification in septic AKI.

## Supplemental Information

10.7717/peerj.20522/supp-1 Supplemental Information 1 Supplementary materials

10.7717/peerj.20522/supp-2 Supplemental Information 2 Raw material

10.7717/peerj.20522/supp-3 Supplemental Information 3 Analysis of the mediation by CRP (A), WBC (B), and neutrophils (C) of the association of PLR and 28-day mortality; by CRP (D), WBC (E), and neutrophils (F) of the association of PLR and 90-day mortality Abbreviations: PLR, platelet-to-lymphocyte ratio; HR, hazard ratio; 95% CI, 95% confidence intervals; CRP, C-reactive protein; WBC, white blood cell.

## Additional Information and Declarations

### Data Availability

The following information was supplied regarding data availability: The raw data are available at figshare: - Zhao, Yuliang (2025). West China Hospital_Figshare.xlsx. figshare. Dataset. https://doi.org/10.6084/m9.figshare.29368754 (Chinese). - Zhao, Yuliang (2025). West China Hospital_Figshare.xlsx. figshare. Dataset. https://doi.org/10.6084/m9.figshare.29368754.v1 (English).
