# Supplementary material for: Platelet-lymphocyte ratio and its dynamic changes predict mortality in septic acute kidney injury patients: a retrospective multi-center study using U.S. database and Chinese hospital data
Source: PeerJ. 2026 Jan 6;14:e20522. doi: 10.7717/peerj.20522 (PMC12786120; doi:10.7717/peerj.20522)
Supplement: Supplemental Information 1 [file peerj-14-20522-s001.docx]

Supplementary Material

**SUPPLEMENTARY APPENDIX**

**Table S1**. The baseline characteristics of participants from training and internal test cohorts.

**Table S2.** The baseline characteristics of participants in the High PLR/ΔPLR group and the Low PLR/ΔPLR group.

**Figure S1.** Analysis of the mediation by CRP (A), WBC (B), and neutrophils (C) of the association of PLR and 28-day mortality; by CRP (D), WBC (E), and neutrophils (F) of the association of PLR and 90-day mortality.

Abbreviations: PLR, platelet-to-lymphocyte ratio; HR, hazard ratio; 95% CI, 95% confidence intervals; CRP, C-reactive protein; WBC, white blood cell.

**Table S1**. The baseline characteristics of participants from training and internal test cohorts.

|  | **Total (n = 1285)** | **Training (n = 899)** | **Test (n = 386)** | **Statistic** | **P** |
| --- | --- | --- | --- | --- | --- |
| Age, year | 65.00 (54.00, 73.00) | 64.00 (53.00, 73.00) | 65.00 (56.00, 73.00) | Z=-0.41 | 0.685 |
| Gender, n(%) |  |  |  | χ²=0.07 | 0.793 |
| Female | 499 (38.83) | 347 (38.60) | 152 (39.38) |  |  |
| Male | 786 (61.17) | 552 (61.40) | 234 (60.62) |  |  |
| BMI, kg/m2 | 29.71 (25.32, 35.11) | 29.74 (25.41, 35.10) | 29.64 (24.92, 35.05) | Z=-0.42 | 0.675 |
|  |  |  |  |  |  |
| **Serum laboratory test** |  |  |  |  |  |
| Hemoglobin, g/L | 10.00 (8.50, 11.70) | 10.00 (8.50, 11.70) | 10.00 (8.43, 11.70) | Z=-0.01 | 0.989 |
| WBC, K/uL | 11.60 (7.60, 17.50) | 11.70 (7.80, 17.70) | 11.10 (7.00, 17.00) | Z=-1.31 | 0.190 |
| Neutrophils, K/uL | 9.23 (5.71, 14.41) | 9.42 (5.82, 14.45) | 8.75 (5.30, 14.33) | Z=-1.23 | 0.220 |
| Lymphocytes, K/uL | 0.88 (0.51, 1.46) | 0.89 (0.53, 1.47) | 0.82 (0.44, 1.41) | Z=-1.59 | 0.111 |
| Platelets, K/uL | 192.00 (125.00, 282.00) | 189.00 (123.00, 282.00) | 198.00 (129.25, 286.75) | Z=-0.88 | 0.381 |
| Urea nitrogen, mg/dL | 30.00 (18.00, 49.00) | 31.00 (18.00, 49.50) | 30.00 (17.00, 47.00) | Z=-0.97 | 0.330 |
| Total protein, g/dL | 5.60 (5.00, 6.30) | 5.60 (4.90, 6.30) | 5.60 (5.00, 6.23) | Z=-0.15 | 0.884 |
| Albumin, g/dL | 2.80 (2.30, 3.20) | 2.70 (2.30, 3.20) | 2.80 (2.40, 3.20) | Z=-0.97 | 0.331 |
| Total bilirubin, umol/L | 10.26 (5.13, 22.23) | 10.26 (6.84, 23.94) | 10.26 (5.13, 18.81) | Z=-1.63 | 0.102 |
| Triglycerides, mmol/L | 0.00 (0.00, 1.81) | 0.00 (0.00, 1.79) | 0.00 (0.00, 1.84) | Z=-1.03 | 0.303 |
| Creatinine, mg/dL | 1.40 (1.00, 2.10) | 1.40 (1.00, 2.15) | 1.40 (0.93, 2.10) | Z=-0.81 | 0.418 |
|  |  |  |  |  |  |
| **Comorbidities** |  |  |  |  |  |
| Hypertension, n(%) | 391 (30.43) | 280 (31.15) | 111 (28.76) | χ²=0.73 | 0.393 |
| Received Transplant, n(%) | 18 (1.40) | 14 (1.56) | 4 (1.04) | χ²=0.53 | 0.466 |
| Diabetes Mellitus, n(%) | 307 (23.89) | 209 (23.25) | 98 (25.39) | χ²=0.68 | 0.409 |
| Coronary Artery Disease, n(%) | 296 (23.04) | 209 (23.25) | 87 (22.54) | χ²=0.08 | 0.782 |
| ARDS, n(%) | 320 (24.90) | 220 (24.47) | 100 (25.91) | χ²=0.30 | 0.586 |
| Pneumonia, n(%) | 291 (22.65) | 208 (23.14) | 83 (21.50) | χ²=0.41 | 0.521 |
| COPD, n(%) | 139 (10.82) | 96 (10.68) | 43 (11.14) | χ²=0.06 | 0.807 |
| Tumor, n(%) | 197 (15.33) | 126 (14.02) | 71 (18.39) | χ²=3.99 | 0.046 |
| UTI, n(%) | 280 (21.79) | 194 (21.58) | 86 (22.28) | χ²=0.08 | 0.780 |
| SLE, n(%) | 9 (0.70) | 8 (0.89) | 1 (0.26) | χ²=0.77 | 0.380 |
| CKD, n(%) | 181 (14.09) | 120 (13.35) | 61 (15.80) | χ²=1.34 | 0.246 |

Abbreviations: MIMIC, Medical Information Mart for Intensive Care; BMI, body-mass index; WBC, white blood cell; ARDS, acute respiratory disease syndrome; COPD, chronic obstructive pulmonary disease; UTI, urinary tract infection; SLE, Systemic lupus erythematosus; CKD, chronic kidney disease.

**Table S2.** The baseline characteristics of participants in the High PLR/ΔPLR group and the Low PLR/ΔPLR group.

| **Variables** | **PLR** | | | | |  | **ΔPLR** | | | | |
| --- | --- | --- | --- | --- | --- | --- | --- | --- | --- | --- | --- |
|  | **Total (n = 899)** | **High PLR (n = 256)** | **Low PLR (n = 643)** | **Statistic** | **P** |  | **Total (n = 627)** | **High ΔPLR (n = 519)** | **Low ΔPLR (n = 108)** | **Statistic** | **P** |
| **Age, year** | 64.00 (53.00, 73.00) | 66.00 (57.00, 75.00) | 64.00 (52.00, 73.00) | Z=-2.87 | 0.004 |  | 64.00 (52.00, 72.00) | 63.00 (52.00, 72.50) | 65.00 (50.00, 71.25) | Z=-0.29 | 0.772 |
| **Gender, n(%)** |  |  |  | χ²=1.19 | 0.275 |  |  |  |  | χ²=4.24 | 0.039 |
| Female | 347 (38.60) | 106 (41.41) | 241 (37.48) |  |  |  | 230 (36.68) | 181 (34.87) | 49 (45.37) |  |  |
| Male | 552 (61.40) | 150 (58.59) | 402 (62.52) |  |  |  | 397 (63.32) | 338 (65.13) | 59 (54.63) |  |  |
| **BMI, kg/m2** | 29.74 (25.41, 35.10) | 30.04 (24.14, 35.88) | 29.71 (25.68, 34.78) | Z=-0.49 | 0.624 |  | 29.86 (25.69, 35.12) | 29.71 (25.27, 35.03) | 30.71 (27.46, 36.46) | Z=-1.01 | 0.314 |
|  |  |  |  |  |  |  |  |  |  |  |  |
| **Serum laboratory test** |  |  |  |  |  |  |  |  |  |  |  |
| Hemoglobin, g/L | 10.00 (8.50, 11.70) | 10.00 (8.20, 11.80) | 10.00 (8.60, 11.60) | Z=-0.47 | 0.638 |  | 10.00 (8.50, 11.50) | 10.10 (8.60, 11.75) | 9.50 (7.70, 10.70) | Z=-3.67 | <.001 |
| WBC, K/uL | 11.70 (7.80, 17.70) | 10.60 (6.68, 16.12) | 12.40 (8.10, 18.25) | Z=-2.86 | 0.004 |  | 12.10 (8.00, 17.95) | 11.80 (7.70, 17.80) | 12.90 (9.78, 19.07) | Z=-2.40 | 0.016 |
| Neutrophils, K/uL | 9.42 (5.82, 14.45) | 9.06 (5.45, 14.17) | 9.45 (5.95, 14.49) | Z=-0.56 | 0.575 |  | 9.64 (6.03, 14.45) | 9.42 (5.77, 14.00) | 10.77 (7.62, 16.15) | Z=-2.79 | 0.005 |
| Lymphocytes, K/uL | 0.89 (0.53, 1.47) | 0.43 (0.25, 0.69) | 1.14 (0.78, 1.77) | Z=-17.00 | <.001 |  | 0.94 (0.55, 1.53) | 0.97 (0.54, 1.57) | 0.88 (0.61, 1.36) | Z=-1.18 | 0.236 |
| Platelets, K/uL | 189.00 (123.00, 282.00) | 247.50 (172.00, 365.75) | 165.00 (107.50, 250.00) | Z=-9.25 | <.001 |  | 197.00 (124.50, 292.00) | 176.00 (115.50, 262.00) | 333.50 (232.00, 412.50) | Z=-9.03 | <.001 |
| Urea nitrogen, mg/dL | 31.00 (18.00, 49.50) | 31.00 (19.00, 53.00) | 30.00 (18.00, 49.00) | Z=-0.70 | 0.486 |  | 30.00 (18.00, 50.00) | 29.00 (18.00, 46.00) | 38.00 (18.75, 68.50) | Z=-2.85 | 0.004 |
| Total protein, g/dL | 5.60 (4.90, 6.30) | 5.45 (4.82, 6.20) | 5.70 (5.00, 6.40) | Z=-1.17 | 0.241 |  | 5.80 (5.00, 6.40) | 5.90 (5.10, 6.60) | 5.10 (4.80, 5.80) | Z=-2.98 | 0.003 |
| Albumin, g/dL | 2.70 (2.30, 3.20) | 2.80 (2.30, 3.12) | 2.70 (2.30, 3.20) | Z=-0.02 | 0.981 |  | 2.80 (2.30, 3.20) | 2.80 (2.30, 3.20) | 2.50 (2.20, 2.90) | Z=-3.29 | <.001 |
| Total bilirubin, umol/L | 10.26 (6.84, 23.94) | 8.55 (5.13, 20.52) | 11.97 (6.84, 25.65) | Z=-2.73 | 0.006 |  | 10.26 (5.13, 22.23) | 10.26 (5.99, 23.94) | 6.84 (5.13, 15.39) | Z=-3.01 | 0.003 |
| Triglycerides, mmol/L | 174.00 (109.00, 278.00) | 193.00 (111.25, 280.25) | 168.00 (106.00, 278.00) | Z=-0.85 | 0.396 |  | 171.50 (112.25, 274.50) | 179.00 (114.00, 284.00) | 162.00 (109.00, 265.00) | Z=-0.74 | 0.462 |
| Creatinine, mg/dL | 1.40 (1.00, 2.15) | 1.30 (1.00, 2.40) | 1.40 (1.00, 2.10) | Z=-0.29 | 0.768 |  | 1.40 (1.00, 2.20) | 1.40 (1.00, 2.00) | 1.70 (0.97, 3.05) | Z=-2.27 | 0.023 |
|  |  |  |  |  |  |  |  |  |  |  |  |
| **Comorbidities** |  |  |  |  |  |  |  |  |  |  |  |
| Hypertension, n(%) | 280 (31.15) | 76 (29.69) | 204 (31.73) | χ²=0.35 | 0.551 |  | 213 (33.97) | 174 (33.53) | 39 (36.11) | χ²=0.27 | 0.606 |
| Received Transplant, n(%) | 14 (1.56) | 4 (1.56) | 10 (1.56) | χ²=0.00 | 1.000 |  | 11 (1.75) | 10 (1.93) | 1 (0.93) | χ²=0.10 | 0.750 |
| Diabetes Mellitus, n(%) | 209 (23.25) | 61 (23.83) | 148 (23.02) | χ²=0.07 | 0.795 |  | 143 (22.81) | 119 (22.93) | 24 (22.22) | χ²=0.03 | 0.874 |
| Coronary Artery Disease, n(%) | 209 (23.25) | 67 (26.17) | 142 (22.08) | χ²=1.71 | 0.190 |  | 137 (21.85) | 111 (21.39) | 26 (24.07) | χ²=0.38 | 0.539 |
| ARDS, n(%) | 220 (24.47) | 72 (28.12) | 148 (23.02) | χ²=2.58 | 0.108 |  | 131 (20.89) | 105 (20.23) | 26 (24.07) | χ²=0.80 | 0.371 |
| Pneumonia, n(%) | 208 (23.14) | 60 (23.44) | 148 (23.02) | χ²=0.02 | 0.893 |  | 126 (20.10) | 102 (19.65) | 24 (22.22) | χ²=0.37 | 0.544 |
| COPD, n(%) | 96 (10.68) | 30 (11.72) | 66 (10.26) | χ²=0.41 | 0.524 |  | 60 (9.57) | 52 (10.02) | 8 (7.41) | χ²=0.70 | 0.401 |
| Tumor, n(%) | 126 (14.02) | 39 (15.23) | 87 (13.53) | χ²=0.44 | 0.507 |  | 80 (12.76) | 62 (11.95) | 18 (16.67) | χ²=1.79 | 0.181 |
| UTI, n(%) | 194 (21.58) | 53 (20.70) | 141 (21.93) | χ²=0.16 | 0.687 |  | 140 (22.33) | 113 (21.77) | 27 (25.00) | χ²=0.54 | 0.464 |
| SLE, n(%) | 8 (0.89) | 3 (1.17) | 5 (0.78) | χ²=0.03 | 0.861 |  | 4 (0.64) | 2 (0.39) | 2 (1.85) | - | 0.139 |
| CKD, n(%) | 120 (13.35) | 43 (16.80) | 77 (11.98) | χ²=3.68 | 0.055 |  | 83 (13.24) | 66 (12.72) | 17 (15.74) | χ²=0.71 | 0.399 |

Abbreviations: PLR, platelet-to-lymphocyte ratio; BMI, body-mass index; WBC, white blood cell; ARDS, acute respiratory disease syndrome; COPD, chronic obstructive pulmonary disease; UTI, urinary tract infection; SLE, Systemic lupus erythematosus; CKD, chronic kidney disease.
