# Supplementary material for: Platelet-lymphocyte ratio and its dynamic changes predict mortality in septic acute kidney injury patients: a retrospective multi-center study using U.S. database and Chinese hospital data
Source: PeerJ. 2026 Jan 6;14:e20522. doi: 10.7717/peerj.20522 (PMC12786120; doi:10.7717/peerj.20522)
Supplement: Supplemental Information 2 [file peerj-14-20522-s002.docx]

10.6084/m9.figshare.29368754
