# Supplementary figures and images for: Platelet-lymphocyte ratio and its dynamic changes predict mortality in septic acute kidney injury patients: a retrospective multi-center study using U.S. database and Chinese hospital data
Source: PeerJ. 2026 Jan 6;14:e20522. doi: 10.7717/peerj.20522 (PMC12786120; doi:10.7717/peerj.20522)

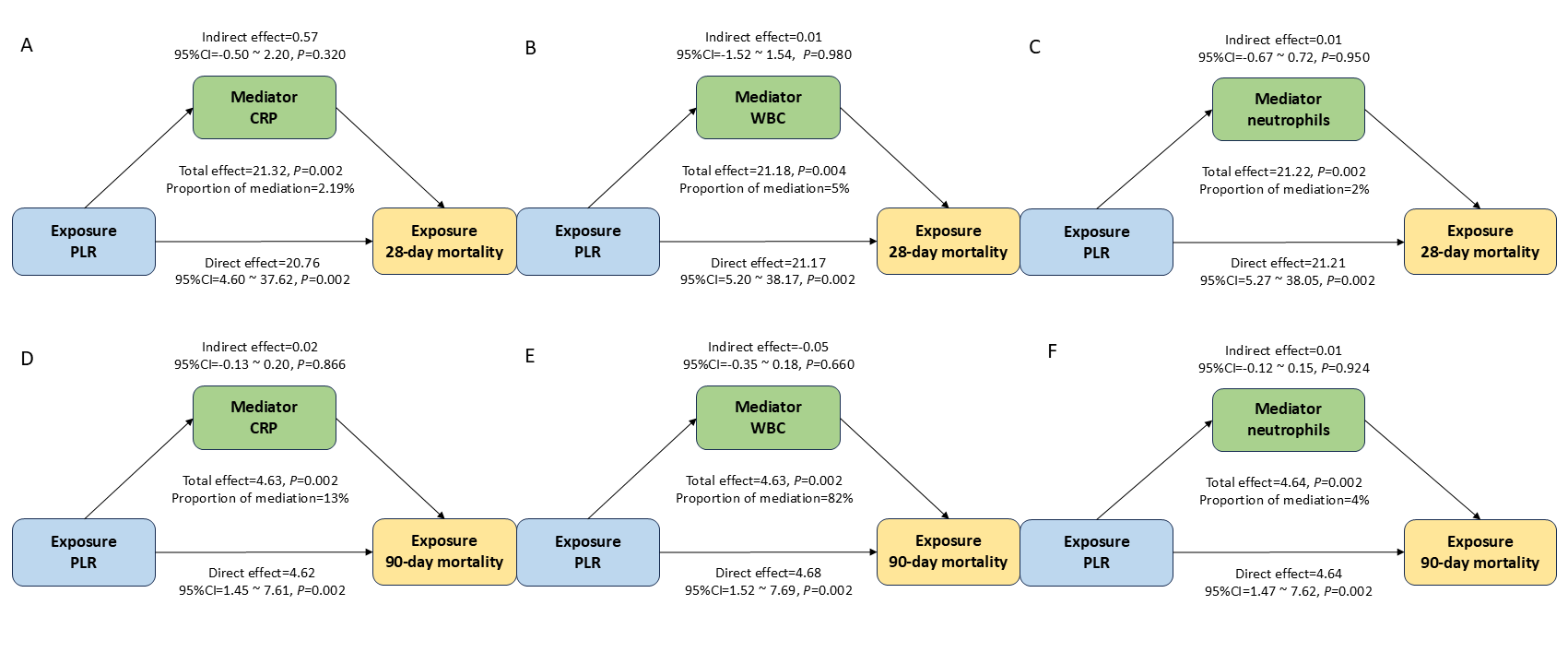

Supplement: Supplemental Information 3 — Abbreviations: PLR, platelet-to-lymphocyte ratio; HR, hazard ratio; 95% CI, 95% confidence intervals; CRP, C-reactive protein; WBC, white blood cell. [file peerj-14-20522-s003.tif]
